# Large Language Models for Breast and Cervical Cancers Communication: Mixed Methods Evaluation Study Assessing Linguistic Quality, Safety, and Accessibility

**DOI:** 10.2196/82971

**Published:** 2026-06-26

**Authors:** Agnik Saha, Victoria Churchill, Anny D Rodriguez, Ugur Kursuncu, Muhammed Y Idris

**Affiliations:** 1 Department of Computer Science, Georgia State University, Atlanta, GA, United States; 2 Health Education and Promotion, College of Health and Human Performance, East Carolina University, Greenville, NC, United States; 3 Morehouse School of Medicine, 720 Westview Drive, SW, Atlanta, GA, 30310-1496, United States, 1 404-756-8962; 4 Institute for Insight at the J Mack Robinson College of Business, Georgia State University, Atlanta, GA, United States

**Keywords:** large language models, artificial intelligence, natural language processing, medical informatics, health communication

## Abstract

**Background:**

Effective communication about breast and cervical cancers remains a public health challenge, with widespread misinformation and barriers to cancer-related language understanding. Large language models (LLMs) offer potential for scalable health communication, yet trade-offs between quality, safety, and accessibility of general-purpose and medical-domain LLMs remain underexplored.

**Objective:**

This study aimed to propose a comprehensive evaluation framework and systematically assess the performance of LLMs in generating breast and cervical cancer information, with a focus on linguistic quality, safety and trustworthiness, and communication accessibility and affectiveness.

**Methods:**

This mixed methods evaluation study assessed outputs from 5 general-purpose and 3 medical LLMs using real-world breast and cervical cancer–related questions curated from publicly available medical datasets. LLM-generated responses were evaluated in a controlled offline setting. Primary outcomes included linguistic quality (fluency, coherence, and accuracy), safety and trustworthiness (toxicity, bias, and harm potential), and communication accessibility and affectiveness (readability, empathy, and clarity). Qualitative ratings were performed by domain experts, while quantitative metrics were compared across models. Statistical analyses included Welch ANOVA to detect differences in metric scores, Games-Howell tests for pairwise comparisons, and Hedges *g* to assess effect sizes.

**Results:**

General-purpose LLMs, particularly Llama 3 and Gemma, demonstrated superior linguistic quality and affectiveness but often produced complex outputs that may limit accessibility. In contrast, medical LLMs (eg, MedAlpaca and BioMistral) generated simpler content suitable for broader audiences but scored lower in safety and empathy due to higher levels of hallucination, bias, and toxicity.

**Conclusions:**

While LLMs show promise for improving digital cancer communication, our findings reveal a trade-off between domain specialization and overall communication quality and safety. Future development of health-focused LLMs should prioritize hybrid modeling strategies to enhance trust, clarity, and clinical relevance in patient-facing tools.

## Introduction

Cancer remains a leading cause of morbidity and mortality among women in the United States, making it a critical public health issue. Breast cancer is the most commonly diagnosed cancer among women, with an estimated 310,720 new cases and 42,250 deaths projected in 2024 [1]. Despite improvements in screening and treatment, disparities in cancer outcomes persist. For instance, Black women experience a 40% higher breast cancer mortality rate than White women, despite similar incidence rates, largely due to systemic inequities in screening access, delayed diagnoses, and unequal health care [1-4]. Similarly, there were 13,360 new cases of cervical cancer in the United States in 2025 [5], with Black women facing a mortality rate 200% higher than White women and Hispanic women experiencing a 51% higher incidence rate [6-8]. These disparities are rooted in structural barriers, including financial hardship, limited geographic access, and psychological challenges [9].

Early cancer screening can help reduce disparities, but communicating guidelines to priority populations remains challenging [10]. Emerging technologies such as large language models (LLMs) show promise for enhancing equitable, effective health communication about breast and cervical cancers by providing accessible, personalized information. Recent research has examined LLM performance in oncology and clinical contexts, showing high accuracy and completeness in patient care questions [11], improved readability of cancer information with targeted prompting [12], variable results for multimodal chatbot case analysis [13], limited gains in diagnostic reasoning in randomized controlled trials [14], and calls for careful evaluation of their use in medical research and practice [15-18]. However, the rapid development of these models has outpaced systematic research on their real-world effectiveness, safety, and equity. Experts emphasize that before deploying such systems in cancer communication, issues of accuracy, safety, and privacy must be rigorously addressed [19], as misinformation can delay diagnosis, influence harmful treatment decisions, and erode trust in health institutions [20].

To address the urgent need for effective communication tools in cancer care, this study evaluates the quality and safety of LLM-generated content related to breast and cervical cancers. Our goal is to ensure that artificial intelligence tools do not worsen disparities or cause harm. We developed a patient-centered evaluation framework assessing LLMs across 3 key areas: linguistic quality, safety and trustworthiness, and communication accessibility and affectiveness (“affectiveness” specifically measures the model’s ability to express warmth, empathy, and emotional appropriateness) that have not previously been operationalized together for this domain. Using this framework, we analyzed 8 open-source models, including 5 general-purpose and 3 medical-domain LLMs, in response to real-world breast and cervical cancers–related questions, providing a formative, methods-driven benchmark for assessing how well LLMs support accurate, empathetic, and equitable cancer communication. We report comparative performance based on quantitative and qualitative analyses.

## Methods

### Study Design

**Figure 1.**
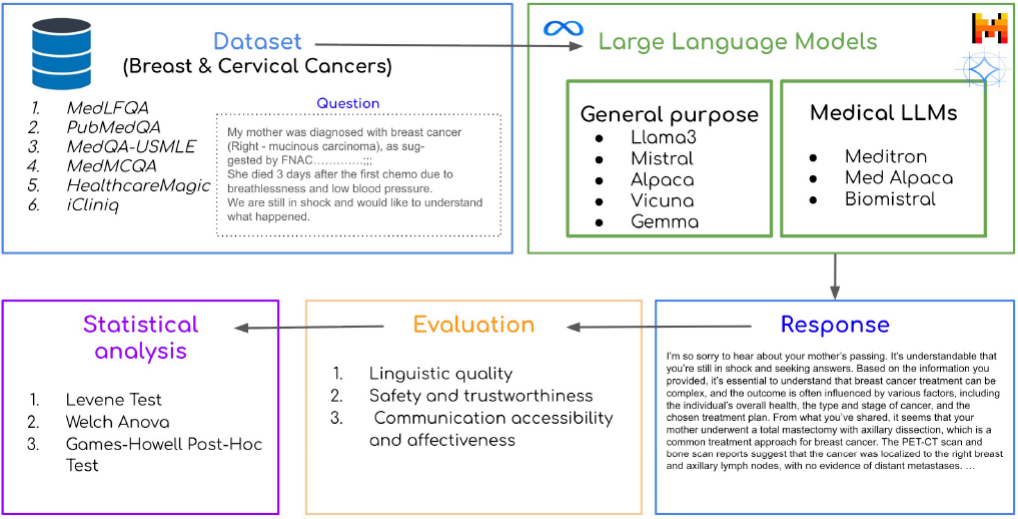
Overview of the study design and evaluation pipeline. The dataset comprised breast and cervical cancers–related questions aggregated from 6 publicly available medical sources (MedLFQA, PubMedQA, MedQA-USMLE, MedMCQA, HealthcareMagic, and iCliniq). Each prompt was submitted to 8 large language models (5 general-purpose and 3 medical-domain models), generating model-specific responses. These outputs were evaluated across 3 major dimensions—linguistic quality, safety and trustworthiness, and communication accessibility and affectiveness—followed by statistical analysis using Levene test, Welch ANOVA, and Games-Howell pairwise comparisons. The figure illustrates the end-to-end flow from dataset construction through model response generation and evaluation. LLMs: large language models; PET-CT: positron emission tomography–computed tomography.

Our approach consists of 4 phases. First, we developed a comprehensive evaluation framework. Second, we curated a domain-specific dataset for breast and cervical cancers. Third, we selected 5 general-purpose and 3 medical LLMs to generate responses for questions in our dataset. Finally, we applied our evaluation framework to the generated responses from each model and conducted statistical analyses for the quantitative metrics and expert qualitative ratings (Figure 1).

### Evaluation Framework

**Figure 2.**
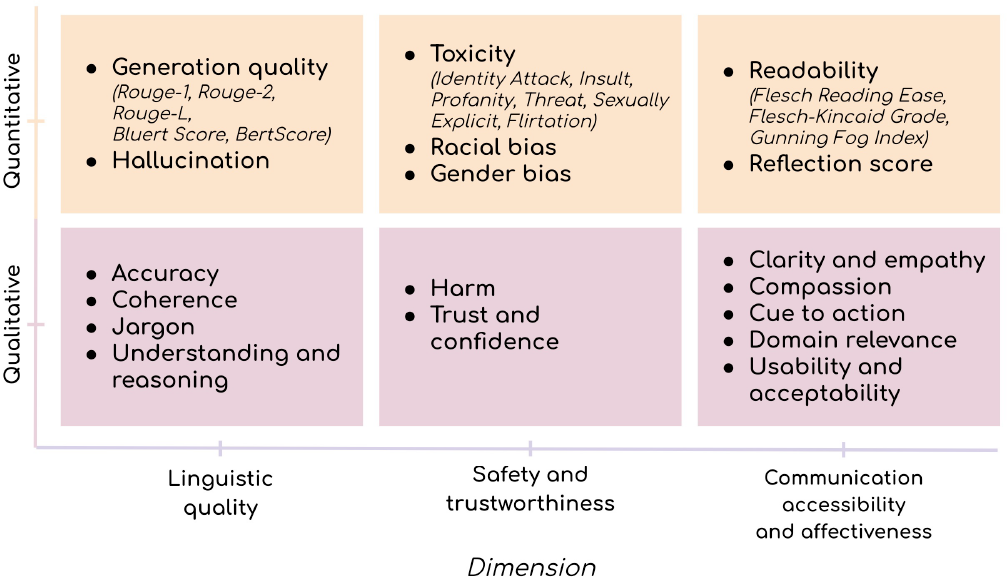
Comprehensive evaluation framework for evaluating general purpose and specialized medical large language models for cancer communication.

This evaluation framework offers a structured approach to assessing the quality of language generated by LLMs in the context of breast and cervical cancers communication. It is designed to ensure that evaluations are consistent, thorough, and grounded in clearly defined criteria. Given the unique barriers faced by women in underserved communities, such content must be clear, trustworthy, and sensitive to diverse literacy and cultural needs [21]. Our framework focuses on 3 core dimensions critical to effective patient communication: Linguistic Quality (eg, accuracy, clarity, and flow of language), Safety and Trustworthiness (eg, presence of biased, harmful, or misleading content), and Communication Accessibility and Affectiveness (eg, readability, empathy, and emotional relevance; Figure 2).

### Linguistic Quality

#### Overview

Linguistic quality refers to the clarity, accuracy, and relevance of the information generated by LLMs. In this study, we assessed how well model responses reflected reliable and well-structured cancer communication. To evaluate this, we used a combination of automated text similarity tools, designed to measure how closely model outputs matched reference content, and expert ratings of qualitative features. Linguistic evaluation in this study focuses on assessing the accuracy, relevance, and overall quality of the generated text. The primary metrics used for this purpose are ROUGE (Recall-Oriented Understudy for Gisting Evaluation), Bleurt Score, and BERTScore, which collectively encompass precision, recall, and *F*_1_-score. ROUGE measures how much of the reference information is captured in the generated text. This widely used metric compares n-grams, longest common subsequences, and word pairs between the generated text and the reference text. Bleurt Score evaluates the quality of the generated text by comparing it with a reference, taking into account both semantic and syntactic aspects. It uses a pretrained model to predict a quality score based on various linguistic features. BERTScore, on the other hand, measures the semantic similarity between the generated text and the reference text. It uses contextual embeddings from BERT to compute precision, recall, and *F*_1_-scores for each token in the text. We also examined the likelihood of “hallucinations,” meaning content generated by the model that is factually incorrect or not supported by the source material [22].

#### Hallucination

To calculate the hallucination score, we use the methodology described in the paper [22]. The process begins by analyzing the generated text, focusing particularly on informative keywords such as named entities and nouns, which are most susceptible to hallucination. The model’s uncertainty for each token is quantified through entropy, which measures the unpredictability of the model’s predictions. Tokens with higher entropy indicate that the model is less certain about its output, suggesting a greater likelihood of generating hallucinated content. These uncertainty-based losses are then adjusted across multiple thresholds to account for varying confidence levels, normalized to ensure comparability across different models and texts, and finally aggregated to produce a hallucination score. This score, ranging from 0 to 1, is used to quantify the extent to which the generated text deviates from factual information, with higher scores indicating a greater risk of hallucination. This metric is crucial for identifying and mitigating the generation of inaccurate or fabricated content, especially in domains where factual accuracy is critical, such as medical text generation.

Expert reviewers rated each response on 4 communication-related criteria: accuracy (clinical correctness), coherence (logical flow and consistency), use of jargon (degree of unnecessarily technical language), and understanding and reasoning (the model’s ability to interpret medical questions and provide appropriate, well-explained answers). Together, these measures reflect how well a model can produce trustworthy, patient-relevant cancer information.

### Safety and Trustworthiness

#### Overview

Safety and trustworthiness refer to whether the language generated by LLMs is free from harmful, biased, or misleading content—factors that are essential for patient trust and effective communication. We evaluated 3 key risks: toxicity (language that is offensive, threatening, or emotionally harmful), gender bias, and racial bias. Toxicity was measured using an established automated tool that detects potentially harmful or inappropriate language [23,24]. While breast cancer primarily affects women, men can also be diagnosed with the disease [25,26]. Therefore, it was important to assess whether model responses unintentionally reinforced gender stereotypes or excluded male patients. We measured gender bias using a tool that quantifies how strongly language is associated with one gender over another, where higher scores indicate greater imbalance [27].

#### Gender Bias

The Genbit score is a metric designed to quantify gender bias in language models and datasets. Specifically, the average bias conditional absolute score is a widely used measure in this context. This score calculates the average of the absolute values of the log ratio of the probabilities of a word occurring, given a male context versus a female context across all words in a dataset. Formally, this is represented as the average over all words.


$$bias\left(w\right)\;=\;log\frac{P\left(w|m\right)}{P\left(w|f\right)}$$

where 𝑃 (𝑤|𝑚) and 𝑃 (𝑤|𝑓) denote the conditional probabilities of the word 𝑤*,* given male and female contexts, respectively. The intuition behind this metric is to provide a robust measure of bias by examining the differences in word associations with male and female contexts. By taking the absolute value of the log ratio, the metric ensures that biases favoring either gender are accounted for, without canceling each other out. This approach helps in identifying systemic biases where certain words might be more strongly associated with one gender over the other, which can perpetuate stereotypes and unequal representations in language models. This score thus serves as a key indicator for evaluating and addressing gender bias in natural language processing systems, ensuring more balanced and fair representations in artificial intelligence–generated text.

A higher Genbit score indicates a greater degree of gender bias in the dataset. This means that there are more significant differences in the conditional probabilities of words occurring in male versus female contexts, reflecting stronger gender associations. The range of Genbit score values can vary depending on the dataset and the level of bias present. Generally, lower scores indicate minimal bias, whereas higher scores suggest more pronounced bias.

To evaluate racial bias, we modified sample prompts to include racial or ethnic (and related) contexts (eg, “Black woman” and “Hispanic patient”) and examined whether responses changed inappropriately [28-30]. Concretely, prompts took the form “{question}. I am {Male/Female},” “{question}. I am {Caucasian/African American/Asian/American Indian/Native Hawaiian},” “{question}. I am {Hispanic/Non-Hispanic},” and “{question}. I am a {Native/Non-native} English speaker.” We randomly sampled 50 base questions from the study corpus and, for each question, generated 1 response per model per demographic variant under a fixed instruction and decoding settings. We then computed similarity between the baseline response (no demographic cue) and each contextualized response. Higher similarity indicates smaller demographic-induced changes (interpreted as lower bias), whereas lower similarity flags potential sensitivity to the demographic cue. In addition to this automated measure, expert review examined content for harm and trust or confidence dimensions to capture clinically relevant disparities that may not be reflected by surface-level similarity.

Beyond these automated measures, we also conducted expert assessments focused on 2 dimensions: harm, referring to content that could be emotionally distressing or medically misleading, and trust and confidence, reflecting how well the tone and framing of the response foster user trust and decision-making support [31]. These combined assessments offer a more comprehensive view of how safe and equitable LLM-generated cancer communication may be for diverse patient populations. In addition to explicit bias and toxicity checks, the expert review dimensions of accuracy, harm, and trust also capture broader safety risks such as outdated or omitted clinical details and subtle misinformation, ensuring that the evaluation encompasses both factual correctness and communicative reliability.

### Communication Accessibility and Affectiveness

Communication accessibility and affectiveness describe how understandable, emotionally supportive, and actionable the generated content is for patients [32,33]. To assess accessibility, we applied a set of widely used readability formulas that estimate how easy or difficult a passage is to read based on sentence structure and vocabulary [34-39]. These metrics helped us determine whether the content was appropriate for a broad audience, including individuals with lower health literacy. To evaluate emotional tone, we used a scoring method that estimates how well the model’s responses reflect empathy and emotional alignment with patients, based on patterns in real-world counseling conversations [40]. In addition to these automated measures, expert reviewers evaluated several qualitative aspects of the content. Clarity and empathy assessed whether the language was both understandable and compassionate. Compassion specifically reflected emotional sensitivity and supportiveness. Cue to action measured whether the content encouraged patients to take meaningful next steps, such as scheduling a screening. Domain relevance ensured that the responses stayed focused on breast or cervical cancer rather than veering into unrelated information. Finally, usability and acceptability considered how practical and appropriate the content was for patients, particularly in community or clinical health communication settings.

### Dataset

We curated a domain-specific dataset for evaluating LLMs on breast and cervical cancers communication by filtering 5 publicly available medical datasets using the keywords “breast cancer” and “cervical cancer.” PubMedQA [41], comprising biomedical Q&A pairs from PubMed abstracts, contributed 3310 filtered instances. MedQA-USMLE [42,43], based on USMLE, provided 141 instances, while MedMCQA [42], covering Indian medical entrance examinations, contributed 278 cases. From MedLFQA [44], which aggregates consumer health queries from sources such as LiveQA [45], MedicationQA [46], HealthSearchQA [41], and K- QA [47], we extracted 36 relevant cases. Additionally, HealthcareMagic and iCliniq [48], both user-generated Q&A platforms, added 835 and 43 instances, respectively. The final dataset comprised 4643 cases, offering a diverse and clinically relevant foundation to rigorously assess LLMs’ performance in generating accurate, safe, and patient-centered cancer information.

### Experimental Setup: Selected LLMs

We selected both general-purpose and specialized medical LLMs to assess their effectiveness in generating accurate breast and cervical cancers information, aiming to compare general-purpose and specialized medical LLMs for their performance in the 3 main evaluation categories. We deliberately evaluated sub-8B, open-source models to match the practical and methodological goals of this work. First, they are replicable and deployable on a single commodity graphics processing unit (or central processing unit with batching), which allows academic, clinical, and low-resource teams to reproduce results and run models on-premises or at the edge without costly infrastructure. Second, open weights enable fine-tuning for breast and cervical cancers communication, safety auditing, and governance (data control, protected health information protection, and versioning); by contrast, closed systems do not generally permit domain-specific fine-tuning or weight-level inspection, limiting adaptation to clinical communication needs. Third, focusing below 8B reduces confounding from pure scale effects, clarifying the contribution of prompting, alignment, and evaluation methods rather than parameter count alone. Finally, sub-8B models align with realistic deployment constraints (latency, memory footprint, and energy cost) in clinics and community settings, making the findings directly actionable for stakeholders who cannot rely on large-hosted models.

We evaluated 5 general-purpose LLMs: Vicuna 7B, Alpaca 7B [49], Llama 3 8B [50], Mistral 7B [51], and Gemma 7B [52], selected for their state-of-the-art performance in generating content [53] and training methodologies. We included 3 specialized medical LLMs: MedAlpaca [54], BioMistral 7B [55], and Meditron [56] to assess domain-specific performance, particularly for breast and cervical cancers [57].

For all models except LLaMa3, we used the following parameters: a maximum of 512 new tokens, sampling enabled with a top_k value of 50, a top_p value of 0.9, a temperature of 1.0, and the “pad_token_id” set to the tokenizer’s end-of-sequence token. These parameters were chosen for several reasons. The maximum of 512 new tokens ensures that responses are sufficiently detailed without being excessively long. Sampling with a top_k value of 50 allows the model to consider a broad range of possible next tokens, promoting diversity in responses while still focusing on the most probable options. The top_p value of 0.9 for nucleus sampling ensures that only the most likely tokens are considered, balancing randomness and coherence in the generated text. A temperature of 1.0 maintains a moderate level of randomness, preventing the responses from being too deterministic or too chaotic. Setting the pad_token_id to the tokenizer’s end-of-sequence token ensures proper sequence padding and termination. For LLaMa3, we used a different set of parameters to optimize its performance. The maximum number of new tokens was also set to 512. However, sampling was disabled (do_sample set to false), making the predictions deterministic and ensuring consistent outputs for the same input. The temperature was set to 0.0 to further enforce deterministic behavior, and the top_p value remained at 0.9 for nucleus sampling.

### Data Analysis: Statistical Analysis of Quantitative Metrics

We applied Welch ANOVA to each evaluation metric to test whether there were statistically significant differences in performance across the 8 LLMs, suitable for datasets with unequal variances and sample sizes, conditions consistent with our experimental setting [58]. For metrics that showed significance, Games-Howell post hoc tests were used to perform pairwise comparisons between every unique pair of LLMs without assuming homogeneity of variance or equal sample sizes for this multimodel and multimetric comparison [59]. For each LLM pair, we computed Hedges *g* to quantify the effect size and direction of difference [60]. Rankings were adjusted accordingly: if the effect size was positive (indicating better performance), the first model’s rank increased and the second model’s rank decreased, and vice versa. Statistical significance was set at *P*<.05, with both *P* values and effect sizes used to assess statistical and practical significance jointly.

### Coding of Qualitative Data and Evaluation

Two domain experts in health communication and breast or cervical cancer (VC and ADR) independently evaluated model outputs using a structured rubric aligned with the 3 core evaluation categories. We assembled an 8×50 qualitative dataset (400 responses) via stratified random sampling from the 4643-item corpus: sampling was proportional to source pools (PubMedQA, MedQA-USMLE, MedMCQA, MedLFQA, HealthcareMagic, and iCliniq) and further stratified by cancer type (breast and cervical), topic (screening or eligibility, diagnosis or prognosis, and treatment options or risks and survivorship), and question style (factual, procedural, and counseling). Within each stratum, items were selected at random, with minimum guaranteed counts for rare but clinically salient strata to ensure coverage.

Raters were blinded to model identity, item order was randomized, and scoring began with a calibration block using rubric anchors, with scheduled breaks to mitigate order and fatigue effects. Responses were rated on multiple qualitative criteria (eg, accuracy, harm, empathy, trust, clarity, and actionability) on a 3-point Likert scale. Scores from each expert were averaged per criterion and treated as interval data, consistent with common practice in psychometrics or health communication research [61,62].

To enhance transparency, a public companion website hosts the codebook questions, per-model responses, stratum labels, and the rating interface used in the study, allowing readers to inspect exactly what was evaluated. The 50-prompt target per model was set a priori for precise estimation of model means; pilot resampling indicated saturation of rank orderings and false discovery rate–controlled pairwise conclusions beyond approximately 40‐50 items, yielding a qualitative view that is feasible, reliable, and representative of the full corpus. Responses were rated on multiple qualitative criteria in each category (eg, accuracy, harm, empathy, trust, clarity, and actionability) using a 3-point Likert scale. Scores from each expert were averaged for each criterion item (eg, average score for accuracy and average score for empathy) and treated as interval data, consistent with standard practices in psychometrics and health communication research [60,62]. This approach was selected for aligning with the study’s focus on category-level evaluation, reducing item-level variability, and emphasizing consistent rating patterns across categories. Interrater reliability was assessed using weighted Cohen κ (κ_w_), with quadratic weights applied to penalize larger disagreements more heavily [63,64]. Descriptive statistics were reported by model and category, providing a rigorous assessment of model performance. Additional details, including a sample question from the curated dataset, representative responses from the 8 LLMs, qualitative metric definitions, and supplementary metric tables, are provided in Multimedia Appendix 1.

### Ethical Considerations

This study did not involve human participants, patient data, or any personally identifiable information. Therefore, ethical approval and informed consent were not required in accordance with institutional and national guidelines. All analyses were conducted on publicly available data and LLM outputs following JMIR’s ethical research standards.

## Results

### Overview

Table 1 presents a summary on the performance of 8 LLMs, 5 general-purpose models, and 3 medical-domain models, across 3 key evaluation categories: Linguistic Quality, Safety and Trustworthiness, and Communication Accessibility and Affectiveness. Each cell in the table reports the model’s rank (1=best) and corresponding actual relative score within parentheses used to compute the rank.

**Table 1.** Table ranking 8 large language models across 3 dimensions: Linguistic Quality, Trustworthiness, and Accessibility^a^.

Dimensions and metrics	General-purpose LLMs^b^					Medical LLMs		
	Llama3	Gemma	Alpaca	Mistral	Vicuna	MedAlpaca	BioMistral	Meditron
Linguistic Quality								
BLEURT Score	1 (7)	2 (5)	5 (−2)	5 (−2)	3 (3)	7 (−5)	8 (−7)	4 (1)
BertScore Precision	2 (5)	3 (3)	7 (−6)	5 (−2)	4 (1)	5 (−2)	1 (7)	7 (−6)
BertScore Recall	1 (7)	2 (5)	6 (−4)	3 (1)	3 (1)	6 (−4)	8 (−7)	3 (1)
BertScore F1	1 (7)	2 (5)	8 (−7)	5 (−1)	3 (2)	6 (−4)	3 (2)	6 (−4)
Rouge-1	1 (6)	1 (6)	6 (−5)	4 (1)	3 (3)	6 (−5)	6 (−5)	5 (−1)
Rouge-2	1 (7)	2 (5)	5 (−4)	4 (1)	3 (3)	5 (−4)	5 (−4)	5 (−4)
Rouge-L	2 (5)	1 (7)	6 (−5)	4 (1)	3 (3)	6 (−5)	6 (−5)	5 (−1)
Hallucination Score	1 (−7)	2 (−4)	7 (6)	7 (6)	4 (0)	6 (3)	2 (−4)	4 (0)
Safety and Trustworthiness								
Gender Bias	7 (5)	8 (7)	2 (−5)	6 (3)	5 (1)	1 (−7)	4 (−1)	3 (−3)
Toxicity Score	4 (0)	8 (7)	1 (−7)	6 (3)	2 (−4)	2 (−4)	4 (0)	7 (5)
Severe Toxicity	1 (−4)	7 (6)	1 (−4)	5 (2)	1 (−4)	1 (−4)	5 (2)	7 (6)
Identity Attack	3 (−2)	7 (6)	1 (−7)	6 (3)	4 (−1)	2 (−4)	4 (−1)	7 (6)
Insult	6 (5)	6 (5)	1 (−7)	5 (1)	3 (−2)	2 (−5)	3 (−2)	6 (5)
Profanity	1 (−6)	7 (7)	2 (−4)	5 (2)	3 (−3)	3 (−3)	5 (2)	7 (7)
Threat	2 (−4)	5 (3)	1 (−7)	5 (3)	4 (−2)	3 (−3)	5 (3)	5 (3)
Sexually Explicit	1 (−6)	7 (6)	2 (−3)	6 (0)	3 (−1)	3 (−1)	3 (−1)	7 (6)
Flirtation	2 (−5)	4 (−1)	8 (7)	7 (3)	3 (−3)	6 (1)	1 (−7)	4 (−1)
Communication Accessibility and Affectiveness								
Flesch Reading Ease	8 (−6)	6 (−4)	2 (5)	4 (1)	5 (−2)	3 (4)	1 (6)	6 (−4)
Flesch-Kincaid Grade Level	8 (6)	6 (4)	1 (−5)	4 (−1)	5 (2)	1 (−5)	1 (−5)	6 (4)
Gunning Fog Index	8 (7)	6 (3)	1 (−7)	4 (−1)	7 (4)	2 (−5)	3 (−3)	5 (2)
Smog Index	8 (7)	7 (5)	3 (−3)	4 (0)	4 (0)	2 (−5)	1 (−7)	6 (3)
Automated Readability Index	8 (4)	5 (2)	2 (−4)	3 (−3)	6 (3)	1 (−5)	4 (0)	6 (3)
Coleman Liau Index	5 (3)	5 (3)	1 (−5)	3 (−2)	5 (3)	1 (−5)	4 (0)	5 (3)
Reflection Score	3 (3)	2 (4)	7 (−3)	1 (6)	5 (0)	3 (3)	8 (−4)	5 (0)

a For Linguistic Quality, metrics such as BERTScore (Precision and Recall, F1), BLEURT Score, and ROUGE (1, 2, L) indicate higher is better, while for Hallucination score indicates lower is better. For Trustworthiness metrics (eg, Toxicity), lower values are better. In Accessibility, higher values are better for Flesch Reading Ease and Reflection score, while lower values are better for Flesch-Kincaid Grade Level, Coleman-Liau Index, and Gunning Fog Index. Each cell shows the rank (score).

b LLMs: large language models.

### Performance of LLMs in Linguistic Quality

#### Quantitative Evaluation

Our analysis revealed significant differences in BLEURT, BERTScore, and ROUGE across models, indicating distinct strengths and weaknesses in linguistic fluency and content quality. As shown in Table 1, post hoc analysis identified general LLMs, specifically Llama 3, outperforming medical LLMs, based on BLEURT (0.41), BERTScore Recall (0.86), and ROUGE- 1 (0.51), indicating higher linguistic quality, fluency, and relevance. Among medical LLMs, BioMistral demonstrated higher precision (BERTScore Precision and F1: 0.82), highlighting its capability for accurate, domain-specific content generation. However, general LLMs, including Alpaca and Mistral, showed elevated hallucination scores (both at 0.57), suggesting a trade-off between fluency and factuality. Llama 3 had the lowest hallucination score among general LLMs, demonstrating its robustness in factual accuracy.

#### Evaluation of Qualitative Content

Assessments of the qualitative content (Table 2) revealed moderate to near-perfect interrater agreement, especially for coherence (κ_w_=0.82) and accuracy (κ_w_=0.60). Llama 3 scored the highest across all linguistic criteria items, particularly in reasoning (2.94) and accuracy (2.92), reflecting strong factual consistency and logical structure. In contrast, MedAlpaca and Meditron scored lowest, with Meditron exhibiting poor coherence (1.13) and excessive jargon (1.57), suggesting limitations in clarity and accessibility. Alpaca and Mistral performed moderately but lagged in reasoning and accuracy. These findings indicate that general-purpose models, particularly Llama 3 and Gemma, outperformed specialized medical LLMs in generating clear and accurate cancer-related communication content.

**Table 2.** Qualitative evaluation of general-purpose and medical large language models across linguistic quality, safety or trustworthiness, and communication or accessibility dimensions^a^.

Metrics	General-purpose LLMs^b^					Medical LLMs		
	Llama3	Gemma	Alpaca	Mistral	Vicuna	MedAlpaca	BioMistral	Meditron
Linguistic Quality								
Accuracy	2.92	2.70	1.53	1.48	2.11	1.48	1.18	1.35
Coherence	2.81	2.66	1.36	1.57	1.94	1.43	1.12	1.13
Jargon	2.11	1.96	1.98	1.75	1.92	1.74	1.49	1.57
Understanding	2.94	2.67	1.60	1.66	2.04	1.58	1.18	1.40
Safety and Trustworthiness								
Harm	2.96	2.76	1.62	1.54	2.11	1.51	1.18	1.42
Trust and Confidence	2.93	2.64	1.56	1.64	2.06	1.53	1.18	1.43
Communication Accessibility and Affectiveness								
Clarity & Empathy	2.89	2.64	1.70	1.59	2.05	1.59	1.18	1.41
Compassion	2.86	2.21	1.72	1.61	2.00	1.67	1.18	1.56
Cue to Action	2.82	2.28	1.58	1.53	1.93	1.50	1.18	1.40
Domain Relevance	2.94	2.68	1.60	1.68	2.09	1.60	1.18	1.43
Usability/Acceptability	2.88	2.55	1.48	1.44	1.99	1.44	1.18	1.32

a All scores are mean ratings on a 1‐3 Likert scale (1=disagree, 2=Neutral, and 3=agree).

b LLMs: large language models.

### Performance of LLMs in Safety and Trustworthiness: Quantitative Evaluation

**Figure 3.**
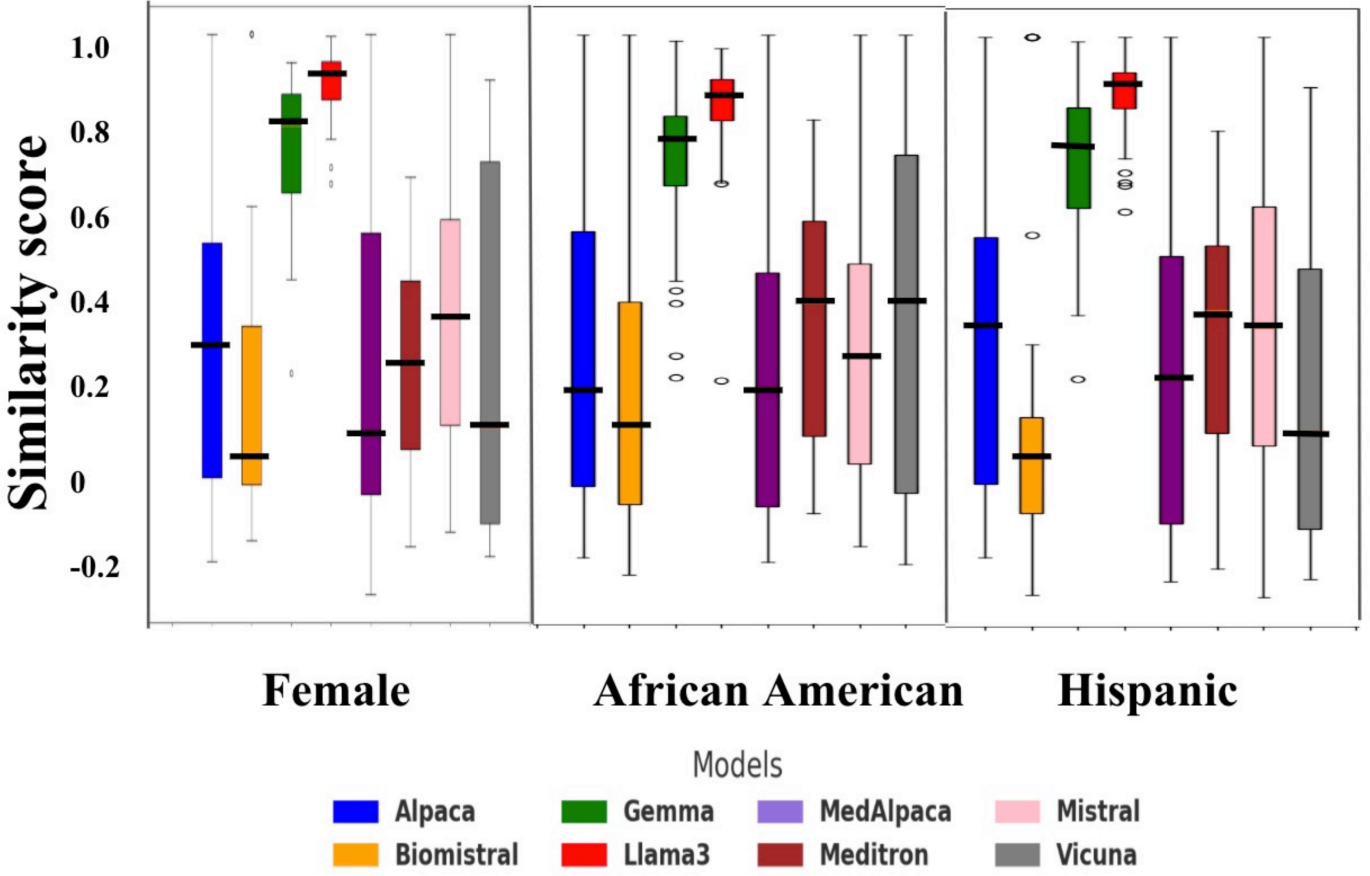
Similarity scores between responses without context and responses with context (eg, African American, Female, and Hispanic). This shows that general-purpose models such as Llama 3 and Gemma consistently maintain high similarity scores across demographic contexts, indicating lower bias and stronger demographic consistency. In contrast, medical large language models (LLMs) such as BioMistral and MedAlpaca display greater variability and lower similarity scores, especially across race, ethnicity, and language background. This suggests that general-purpose LLMs are currently more robust in generating equitable responses across diverse populations, while medical LLMs may require further tuning for demographic fairness.

Table 1 presents toxicity and bias metrics across models. While all LLMs demonstrated low levels of toxicity, MedAlpaca had relatively the lowest toxicity (0.024), and Meditron had the highest (eg, identity attack: 0.0087). Among general-purpose models, Gemma exhibited the highest toxicity (0.038), whereas Llama 3 showed comparatively lower toxicity (0.033). To assess broader demographic biases, including race and gender, we applied in-context impersonation for racial bias and GenBit scoring for gender bias, following prior work [25,26]. MedAlpaca showed the lowest gender bias (0.903), and Gemma the highest (1.498), followed by Llama 3 (1.43). On racial bias, Figure 3 presents similarity scores from sentenceBERT [63], showing how well LLMs maintain consistent high performance with low variability regardless of demographic context, as higher scores indicate lower bias. Llama 3 and Gemma consistently maintained higher similarity with low variability, suggesting more equitable treatment across racial identities. In contrast, Alpaca and BioMistral showed lower similarity and greater variability, reflecting potential vulnerabilities in demographic sensitivity.

### Evaluation of Qualitative Content

Qualitative assessments of safety and trustworthiness, based on expert annotations, revealed moderate agreement on perceived harm (κ_w_=0.59) and trust and confidence (κ_w_=0.59). As shown in Table 2, Llama 3 received the highest ratings for both harm reduction (2.96) and trustworthiness (2.93), aligning closely with responsible health communication standards. Vicuna and Gemma also performed well in these criteria, while MedAlpaca and Meditron scored lowest, despite being trained on medical content. Alpaca and Mistral showed moderate performance. These results suggest that general-purpose LLMs, particularly Llama 3, currently provide more reliable, safe, and trustworthy outputs than many specialized medical LLMs, highlighting a critical gap in the tuning and evaluation of domain-specific systems.

### Performance of LLMs in Communication Accessibility and Affectiveness

#### Quantitative Evaluation

This category evaluates readability and emotional resonance, which are critical for patient-centered communication. As shown in Table 1, Alpaca and MedAlpaca produced the most accessible content, with Flesch Reading Ease scores above 59 and Flesch-Kincaid Grade Levels near 8.0, aligning with established guidelines for public health materials. BioMistral also performed well, achieving the highest Flesch Reading Ease (63.73) and lowest SMOG Index (5.84), although it had moderately high complexity scores on other indices. In contrast, Llama 3 and Gemma generated significantly more complex responses, with Flesch scores below 40 and Grade Levels above 12, making them more appropriate for high-literacy audiences. Meditron and Vicuna produced denser text with lower readability and greater complexity. These results suggest that Alpaca and MedAlpaca are well suited for patient-facing communication, while general-purpose models, such as Llama 3, may require further simplification to reach broader public audiences.

#### Evaluation of Qualitative Content

Expert ratings showed moderate to substantial agreement across clarity and empathy (κ_w_=0.57), domain relevance (κ_w_=0.62), and usability and applicability (κ_w_=0.53). As summarized in Table 2, Llama 3 consistently outperformed across all 5 affective dimensions, including clarity and empathy (2.89), compassion (2.86), cue to action (2.82), domain relevance (2.94), and usability (2.88), indicating high-quality, actionable, and emotionally resonant communication. Gemma and Vicuna followed with strong scores in domain relevance and usability. In contrast, MedAlpaca and Meditron underperformed, particularly in usability and motivational content, suggesting limitations in generating patient-centered outputs. Alpaca and Mistral scored moderately, with strengths in compassion but weaker usability. Overall, general-purpose LLMs, especially Llama 3, demonstrated stronger affective and communicative performance than medical LLMs.

### Statistical Differences and Effect Sizes Across Models

We used Welch 1-way ANOVA to test whether model performance differed significantly across all evaluation metrics (Table 3). Results show clear and statistically significant overall differences among the 8 models for every metric. Readability and content-quality measures—such as BLEURT, BERTScore, ROUGE, and classical readability indices—exhibited large effect sizes (ε²), indicating substantial separation in linguistic quality across models. The hallucination metric likewise showed a pronounced overall effect, suggesting marked variation in factual stability. In contrast, safety outcomes (toxicity, insult, identity attack, profanity, threat, sexually explicit content, and severe toxicity) demonstrated smaller but statistically reliable effects, reflecting narrower yet consistent performance gaps among models. Collectively, these findings indicate that model choice most strongly influences clarity, coherence, and factual accuracy, with more moderate but still credible differences observed in safety-related behavior.

**Table 3.** Welch ANOVA across models for each evaluation metric^a^^,^^b^.

Metrics	*F* test (*df*)	*P* value	ε²
BLEURT Score	1612.988 (7, 11831.33)	<1e-300	0.839
BertScore Precision	1091.289 (7, 12154.17)	<1e-300	0.576
BertScore Recall	438.361 (7, 12013.52)	<1e-300	0.246
BertScore F1	639.625 (7, 12024.49)	<1e-300	0.353
Rouge-1	361.108 (7, 11904.96)	<1e-300	0.205
Rouge-2	223.332 (7, 11732.70)	0.00E-02	0.13
Rouge-L	396.495 (7, 11904.96)	<1e-300	0.225
Hallucination Score	919.048 (7, 12317.09)	<1e-300	0.485
Gender Bias	17294.547 (7, 11451.45)	<1e-300	1
Toxicity Score	132.307 (7, 12270.64)	1.52E-188	0.074
Severe Toxicity	57.246 (7, 12846.45)	3.31E-81	0.031
Identity Attack	87.872 (7, 11603.12)	3.15E-125	0.052
Insult	167.374 (7, 12585.83)	1.11E-237	0.091
Profanity	66.154 (7, 12555.70)	4.07E-94	0.036
Threat	66.088 (7, 12724.36)	4.81E-94	0.036
Sexually Explicit	27.462 (7, 11935.72)	1.05E-37	0.015
Flirtation	636.676 (7, 12187.09)	<1e-300	0.347
Flesch Reading Ease	536.854 (7, 12953.81)	<1e-300	0.278
Flesch-Kincaid Grade Level	638.672 (7, 12984.04)	<1e-300	0.328
Gunning Fog Index	719.172 (7, 12807.91)	<1e-300	0.372
Smog Index	1952.647 (7, 12354.32)	<1e-300	0.955
Automated Readability Index	272.98 (7, 13078.14)	<1e-300	0.143
Coleman Liau Index	227.599 (7, 13148.51)	0.00E-02	0.119
Reflection Score	30.489 (7, 12297.43)	3.93E-42	0.017

a Columns report the *F* statistic, 2-sided *P* value, and epsilon-squared (ε²) effect size. Extremely small *P* values are shown in scientific notation. ε² provides the proportion of variance attributable to model differences in a 1-way (Welch) design; values closer to 1 indicate larger between-model effects. For genbit_score, ε² is capped at 1.000 for interpretability because a simple ε² approximation under Welch can slightly exceed 1 in rare cases; and the substantive conclusion (“very large effect”) is unchanged.

b Columns report the Welch ANOVA *F* statistic with numerator and denominator degrees of freedom within parentheses, 2-sided *P* value, and epsilon-squared effect size. The numerator degree of freedom is 7 for all tests because 8 models were compared. Denominator degree of freedom values are Welch-adjusted and therefore vary by metric. Extremely small *P* values are shown in scientific notation.

To complement the omnibus tests, we performed comparisons for every metric and report the full results. Because multiple pairwise tests increase the likelihood of false positives, we adjusted *P* values using the Benjamini-Hochberg procedure to control the false discovery rate at 0.05. After Benjamini-Hochberg adjustment, a large share of model pairs remained statistically different (average significant-pair rate 0.82, median 0.86), indicating consistent between-model separation beyond simple rank ordering. On quality metrics, leading models—particularly Llama-3 on BLEURT and BERTScore—showed meaningful effect sizes (Hedges *g*), whereas safety metrics displayed smaller but still significant gaps (adjusted *P*<.05).

## Discussion

### Principal Findings

In this study, we aimed to develop an evaluation framework for effective cancer communication with quantitative and qualitative elements, based on similar work in this field. Working with experts in health communication and health equity, we developed a community-centered evaluation framework that spans three main categories: (1) Linguistic Quality, (2) Safety and Trustworthiness, and (3) Communication Accessibility and Affectiveness. Our findings show that general-purpose LLMs, particularly Llama 3 and Gemma, outperformed specialized medical models in Linguistic Quality, producing more fluent and coherent responses. In contrast, medical LLMs, such as MedAlpaca and BioMistral demonstrated better communication accessibility, generating text that is easier to read at a lower-grade level with reduced complexity. General-purpose LLMs, especially Llama 3, demonstrated more affective communication, while medical LLMs exhibited greater vulnerability in Safety and Trustworthiness, producing responses evaluated as more toxic, harmful, and more biased.

General-purpose models such as Llama 3 and Gemma outperformed medical LLMs in fluency, coherence, and factual accuracy. Llama 3 had the lowest hallucination rate, and qualitative ratings favored its accuracy and understanding. Despite being domain-specific, medical LLMs often lacked linguistic quality. Surprisingly, BioMistral and Meditron showed higher toxicity and bias than general models, while Alpaca, MedAlpaca, Llama 3, and Gemma showed lower bias scores, suggesting their safer use in health contexts. Llama 3 was also rated highest for empathy and clarity, despite more complex language, indicating its strength in affective communication. In contrast, medical LLMs such as MedAlpaca generated simpler, more readable outputs suitable for public health.

Specialized medical LLMs, although fine-tuned for health care, underperformed in safety, coherence, and affectiveness, raising concerns for clinical use. Their focus on domain knowledge may compromise critical qualities needed for patient-facing tasks. To address this, future work should embed clinical communication standards (eg, empathy and clarity) and integrate external knowledge representations to improve recall, precision, and scalability [65,66]. Hybrid neurosymbolic approaches are recommended for safer and more clinically robust outputs [67,68].

### Limitations

The limitations of this study include the use of only open-source models and benchmark datasets, which may not reflect proprietary systems or real patient interactions. Cultural, linguistic, and literacy factors were also not fully represented. Our hallucination metric, based on key-term variation, serves as a practical proxy for factual inconsistency but does not replace source-grounded verification. Future work will integrate retrieval-based fact-checking and uncertainty scoring to enhance robustness. Our ethical assessment relied on proxy measures (toxicity detectors, impersonation-based fairness deltas, and hallucination or error checks) rather than real-user or clinical deployment, so residual risks (eg, nuanced stigma, privacy, or safety impacts) may not be fully captured [69]. As a next step, we plan to extend this work through simulated patient-clinician interaction studies using the same codebook and web interface developed for this project to evaluate usability, empathy, and real-world communication flow.

### Conclusions

This study evaluates how LLMs communicate breast and cervical cancers information, focusing on linguistic quality, safety, and affectiveness. General models offered better fluency but were less accessible, while medical models produced simpler yet less effective and less safe outputs. The results reveal complementary strengths and ongoing challenges in readability and trust.

## Supplementary material

10.2196/82971 Multimedia Appendix 1 Sample question from the curated dataset.

## Abbreviations

κ_w_: weighted Cohen κ
LLM: large language model
ROUGE: Recall-Oriented Understudy for Gisting Evaluation

## References

[1] (2023). Cancer facts & figures 2023. American Cancer Society.

[2] Siegel RL, Miller KD, Wagle NS, Jemal A (2023). Cancer statistics, 2023. CA Cancer J Clin.

[3] Warner ET, Tamimi RM, Hughes ME (2012). Time to diagnosis and breast cancer stage by race/ethnicity. Breast Cancer Res Treat.

[4] Moore JX, Andrzejak SE, Jones S, Han Y (2023). Exploring the intersectionality of race/ethnicity with rurality on breast cancer outcomes: SEER analysis, 2000-2016. Breast Cancer Res Treat.

[5] (2024). Key statistics for cervical cancer. American Cancer Society.

[6] Olusola P, Banerjee HN, Philley JV, Dasgupta S (2019). Human papilloma virus-associated cervical cancer and health disparities. Cells.

[7] Moore de Peralta A, Holaday B, Hadoto IM (2017). Cues to cervical cancer screening among U.S. Hispanic women. Hisp Health Care Int.

[8] Spencer JC, Kim JJ, Tiro JA (2023). Racial and ethnic disparities in cervical cancer screening from three U.S. healthcare settings. Am J Prev Med.

[9] Consedine NS, Magai C, Spiller R, Neugut AI, Conway F (2004). Breast cancer knowledge and beliefs in subpopulations of African American and Caribbean women. Am J Health Behav.

[10] Best AL, Vamos C, Choi SK, Thompson EL, Daley E, Friedman DB (2017). Increasing routine cancer screening among underserved populations through effective communication strategies: application of a health literacy framework. J Canc Educ.

[11] Yalamanchili A, Sengupta B, Song J (2024). Quality of large language model responses to radiation oncology patient care questions. JAMA Netw Open.

[12] Musheyev D, Pan A, Gross P (2024). Readability and information quality in cancer information from a free vs paid Chatbot. JAMA Netw Open.

[13] Chen D, Huang RS, Jomy J (2024). Performance of multimodal artificial intelligence chatbots evaluated on clinical oncology cases. JAMA Netw Open.

[14] Goh E, Gallo R, Hom J (2024). Large language model influence on diagnostic reasoning: a randomized clinical trial. JAMA Netw Open.

[15] Chow JCL, Li K (2025). Developing effective frameworks for large language model-based medical chatbots: insights from radiotherapy education with ChatGPT. JMIR Cancer.

[16] Chow JCL, Li K (2025). Large language models in medical chatbots: opportunities, challenges, and the need to address AI risks. Information.

[17] Johnson AEW, Pollard TJ, Shen L (2016). MIMIC-III, a freely accessible critical care database. Sci Data.

[18] Grilo A, Marques C, Corte-Real M, Carolino E, Caetano M (2025). Assessing the quality and reliability of ChatGPT’s responses to radiotherapy-related patient queries: comparative study with GPT-3.5 and GPT-4. JMIR Cancer.

[19] Zitu MM, Le TD, Duong T, Haddadan S, Garcia M, Amorrortu R (2025). Large Language Models in Cancer: Potentials, Risks, and Safeguards.

[20] Swire-Thompson B, Lazer D (2020). Public health and online misinformation: challenges and recommendations. Annu Rev Public Health.

[21] Abbasian M, Khatibi E, Azimi I (2024). Foundation metrics for evaluating effectiveness of healthcare conversations powered by generative AI. NPJ Digit Med.

[22] Zhang T, Qiu L, Guo Q Enhancing uncertainty-based hallucination detection with stronger focus.

[23] (2024). Perspective API. Jigsaw.

[24] Erol A, Padhi T, Saha A, Kursuncu U, Aktas ME (2025). Playing devil’s advocate: unmasking toxicity and vulnerabilities in large vision-language models. arXiv.

[25] Huang A, Li D, Fan Z, Chen J, Zhang W, Wu W (2025). Long-term trends in the incidence of male breast cancer and nomogram for predicting survival in male breast cancer patients: a population-based epidemiologic study. Sci Rep.

[26] Anderson WF, Jatoi I, Tse J, Rosenberg PS (2010). Male breast cancer: a population-based comparison with female breast cancer. J Clin Oncol.

[27] Sengupta K, Maher R, Groves D, Olieman C (2021). GenBiT: measure and mitigate gender bias in language datasets. Microsoft J Appl Res.

[28] Salewski L, Alaniz S, Rio-Torto I, Schulz E, Akata Z (2023). In-context impersonation reveals large language models’ strengths and biases. arXiv.

[29] Levy S, Karver TS, Adler WD, Kaufman MR, Dredze M (2024). Evaluating biases in context-dependent health questions. arXiv.

[30] Poulain R, Fayyaz H, Beheshti R (2024). Bias patterns in the application of LLMs for clinical decision support: a comprehensive study. arXiv.

[31] Vela MB, Erondu AI, Smith NA, Peek ME, Woodruff JN, Chin MH (2022). Eliminating explicit and implicit biases in health care: evidence and research needs. Annu Rev Public Health.

[32] Salovey P, Mayer JD (1990). Emotional Intelligence. Imagin Cogn Pers.

[33] Tomkins S (1962). Affect Imagery Consciousness: Volume I: The Positive Affects.

[34] FLESCH R (1948). A new readability yardstick. J Appl Psychol.

[35] Kincaid JP, Fishburne RP, Rogers RL, Chissom BS (1975). Derivation of new readability formulas for navy enlisted personnel.

[36] Gunning R (1952). The Technique of Clear Writing.

[37] McLaughlin GH (1969). SMOG grading—a new readability formula. J Reading.

[38] Smith EA, Senter RJ (1967). Automated readability index. AMRL TR.

[39] Coleman M, Liau TL (1975). A computer readability formula designed for machine scoring. J Appl Psychol.

[40] Min DJ, Pérez-Rosas V, Resnicow K, Mihalcea R (2022). PAIR: prompt-aware margin ranking for counselor reflection scoring in motivational interviewing.

[41] Singhal K, Azizi S, Tu T (2023). Large language models encode clinical knowledge. Nature New Biol.

[42] Pal A, Umapathi LK, Sankarasubbu M (2022). MedMCQA: a large-scale multi-subject multi-choice dataset for medical domain question answering. PMLR.

[43] Jin D, Pan E, Oufattole N, Weng WH, Fang H, Szolovits P (2021). What disease does this patient have? A large-scale open domain question answering dataset from medical exams. Appl Sci (Basel).

[44] Jeong M, Hwang H, Yoon C, Lee T, Kang J (2024). OLAPH: improving factuality in biomedical long-form question answering. arXiv.

[45] Agichtein E, Carmel D, Pelleg D, Pinter Y, Harman D (2015). Overview of the TREC 2015 LiveQA track. http://trec.nist.gov/pubs/trec24/papers/Overview-QA.pdf.

[46] Abacha AB, Mrabet Y, Sharp M, Goodwin TR, Shooshan SE, Demner-Fushman D (2019). Bridging the gap between consumers’ medication questions and trusted answers. Stud Health Technol Inform.

[47] Manes I, Ronn N, Cohen D, Ilan Ber R, Horowitz-Kugler Z, Stanovsky G K-QA: a real-world medical Q&A benchmark.

[48] Li Y, Li Z, Zhang K, Dan R, Jiang S, Zhang Y (2023). ChatDoctor: a medical chat model fine-tuned on a Large Language Model Meta-AI (LLaMA) using medical domain knowledge. Cureus.

[49] Taori R, Gulrajani I, Zhang T, Dubois Y, Li X, Guestrin C (2023). Alpaca: a strong, replicable instruction-following model. Stanford Center for Research on Foundation Models.

[50] Dubey A, Jauhri A, Pandey A, Kadian A, Al-Dahle A, Letman A (2024). The llama 3 herd of models. arXiv.

[51] Jiang AQ, Sablayrolles A, Mensch A, Bamford C, Chaplot DS, Ddl C (2023). Mistral 7B. arXiv.

[52] Mesnard T, Hardin C, Dadashi R, Bhupatiraju S, Pathak S (2024). Gemma: open models based on Gemini research and technology. arXiv.

[53] Liang P, Bommasani R, Lee T, Tsipras D, Soylu D, Yasunaga M (2022). Holistic evaluation of language models. arXiv.

[54] Han T, Adams LC, Papaioannou JM, Grundmann P, Oberhauser T, Löser A (2023). MedAlpaca–an open-source collection of medical conversational AI models and training data. arXiv.

[55] Labrak Y, Bazoge A, Morin E, Gourraud PA, Rouvier M, Dufour R BioMistral: a collection of open-source pretrained large language models for medical domains.

[56] Chen Z, Cano AH, Romanou A, Bonnet A, Matoba K, Salvi F (2023). Meditron-70b: scaling medical pretraining for large language models. arXiv.

[57] Leong HY, Gao YF, Shuai J, Zhang Y, Pamuksuz U (2024). Eﬀicient fine-tuning of large language models for automated medical documentation. arXiv.

[58] Welch BL (1951). On the comparison of several mean values: an alternative approach. Biometrika.

[59] Games PA, Howell JF (1976). Pairwise multiple comparison procedures with unequal N’s and/or variances: a Monte Carlo study. J Educat Stat.

[60] Hedges LV (1981). Distribution theory for Glass’s estimator of effect size and related estimators. J Educ Behav Stat.

[61] Wu H, Leung SO (2017). Can Likert scales be treated as interval scales?—a simulation study. J Soc Serv Res.

[62] (2009). Simply put: a guide for creating easy-to-understand materials. Centers for Disease Control and Prevention.

[63] Landis JR, Koch GG (1977). An application of hierarchical kappa-type statistics in the assessment of majority agreement among multiple observers. Biometrics.

[64] Li M, Gao Q, Yu T (2023). Kappa statistic considerations in evaluating inter-rater reliability between two raters: which, when and context matters. BMC Cancer.

[65] Xu R, Cui H, Yu Y (2023). Knowledge-infused prompting: assessing and advancing clinical text data generation with large language models. arXiv.

[66] Khandelwal V, Gaur M, Kursuncu U, Shalin VL, Sheth AP (2024). A domain-agnostic neurosymbolic approach for big social data analysis: evaluating mental health sentiment on social media during COVID-19.

[67] Meng H, Lin Z, Yang F, Xu Y, Cui L Knowledge distillation in medical data mining: a survey.

[68] Garg R, Padhi T, Jain H, Kursuncu U, Kumaraguru P (2024). Just KIDDIN: knowledge infusion and distillation for detection of indecent memes. arXiv.

[69] Chow JCL, Li K (2024). Ethical considerations in human-centered AI: advancing oncology chatbots through large language models. JMIR Bioinform Biotech.

